# Glucocorticoid Regulation of Food-Choice Behavior in Humans: Evidence from Cushing's Syndrome

**DOI:** 10.3389/fnins.2016.00021

**Published:** 2016-02-05

**Authors:** Scott J. Moeller, Lizette Couto, Vanessa Cohen, Yelena Lalazar, Iouri Makotkine, Nia Williams, Rachel Yehuda, Rita Z. Goldstein, Eliza B. Geer

**Affiliations:** ^1^Department of Psychiatry, Icahn School of Medicine at Mount SinaiNew York, NY, USA; ^2^Department of Neuroscience, Icahn School of Medicine at Mount SinaiNew York, NY, USA; ^3^Department of Medicine, Icahn School of Medicine at Mount SinaiNew York, NY, USA; ^4^Department of Neurosurgery, Icahn School of Medicine at Mount SinaiNew York, NY, USA

**Keywords:** Cushing's syndrome, choice behavior, decision-making, reward processing, glucocorticoids, cortisol, food craving

## Abstract

The mechanisms by which glucocorticoids regulate food intake and resulting body mass in humans are not well-understood. One potential mechanism could involve modulation of reward processing, but human stress models examining effects of glucocorticoids on behavior contain important confounds. Here, we studied individuals with Cushing's syndrome, a rare endocrine disorder characterized by chronic excess endogenous glucocorticoids. Twenty-three patients with Cushing's syndrome (13 with active disease; 10 with disease in remission) and 15 controls with a comparably high body mass index (BMI) completed two simulated food-choice tasks (one with “explicit” task contingencies and one with “probabilistic” task contingencies), during which they indicated their objective preference for viewing high calorie food images vs. standardized pleasant, unpleasant, and neutral images. All participants also completed measures of food craving, and approximately half of the participants provided 24-h urine samples for assessment of cortisol and cortisone concentrations. Results showed that on the explicit task (but not the probabilistic task), participants with active Cushing's syndrome made fewer food-related choices than participants with Cushing's syndrome in remission, who in turn made fewer food-related choices than overweight controls. Corroborating this group effect, higher urine cortisone was negatively correlated with food-related choice in the subsample of all participants for whom these data were available. On the probabilistic task, despite a lack of group differences, higher food-related choice correlated with higher state and trait food craving in active Cushing's patients. Taken together, relative to overweight controls, Cushing's patients, particularly those with active disease, displayed a reduced vigor of responding for food rewards that was presumably attributable to glucocorticoid abnormalities. Beyond Cushing's, these results may have relevance for elucidating glucocorticoid contributions to food-seeking behavior, enhancing mechanistic understanding of weight fluctuations associated with oral glucocorticoid therapy and/or chronic stress, and informing the neurobiology of neuropsychiatric conditions marked by abnormal cortisol dynamics (e.g., major depression, Alzheimer's disease).

## Introduction

Hypothalamic-pituitary-adrenal (HPA) axis activity is essential for ensuring body homeostasis and survival during stress. Stress initiates a cascade of neuroendocrine responses including secretion of corticotropin-releasing hormone (CRH) from the hypothalamus, which stimulates adrenocorticotrophic hormone (ACTH) secretion from pituitary corticotrophs, culminating in glucocorticoid (GC) secretion from the adrenal glands (Smith and Vale, 2006). This stress response precipitating the release of GCs has downstream effects on multiple regulatory systems such as appetite control (La Fleur, 2006), thereby helping to regulate eating and satiety (Uchoa et al., 2014). Chronic stress, which results in persistent excess GC exposure, has been associated with increased hepatic glucose production, decreased glucose transport and utilization, decreased protein synthesis, enhanced protein degradation in muscles, and adipose tissue expansion, culminating in obesity and insulin resistance (Nieuwenhuizen and Rutters, 2008; Lacroix et al., 2015). However, although GCs regulate food intake and resulting body mass, the mechanisms for these relationships in humans are not well-understood.

One potential mechanism of how GCs orchestrate eating behavior could involve reward processing (Adam and Epel, 2007). This hypothesis follows from research literatures that have examined modulation of reward processing via transiently (experimentally) increased cortisol (e.g., experimental administration of cortisol or laboratory stressors) or chronically enhanced cortisol (e.g., due to early life stress). Studies have reliably shown decreased *non-food* reward processing in individuals exposed to stress. For example, experimental administration of cortisol or laboratory stressors (e.g., cold pressors, watching aversive movie clips, or trying to solve unanswerable math problems) are associated with lower eagerness to receive task-related rewards or lower functional magnetic resonance imaging (fMRI) activity [e.g., during a card-guessing task or the anticipation phase of the monetary incentive delay (MID) tasks] in corticolimbic regions relevant to reward (e.g., striatum, amygdala, hippocampus, and/or medial prefrontal cortex) (Ossewaarde et al., 2011; Porcelli et al., 2012; Montoya et al., 2014). In addition, individuals characterized as highly stress-reactive (i.e., showing high cortisol reactivity and self-reported negative affect after a stress induction) showed diminished sensitivity to reward, but not punishment, under stress (Berghorst et al., 2013). Individuals who experienced childhood maltreatment or early life stress, relative to individuals who did not experience these stressors, also rated reward cues as being less pleasant and showed blunted fMRI response in the globus pallidus, a striatal dopaminergic region, to reward cues during a MID task (Dillon et al., 2009); showed less ventral striatal fMRI response to happy faces (Goff et al., 2013); and responded less to money as an incentive to improve task performance (Mueller et al., 2012). Results of studies examining the effects of stress on *food* reward have been less consistent. A large body of literature suggests that presence of stress typically elevates food reward and consumption, often for unhealthy foods with high caloric densities (Tomiyama et al., 2011; Groesz et al., 2012; Hoffman et al., 2012; Talbot et al., 2013; Tryon et al., 2013; Aschbacher et al., 2014; Ferreira de Sa et al., 2014; Pursey et al., 2014; Maier et al., 2015). However, one study in healthy individuals showed that stress was associated with decreased activation in the reward circuitry including the orbitofrontal cortex and putamen specifically during a food-choice procedure (i.e., when participants selected foods to be consumed later) (Born et al., 2010). Moreover, animal models (typically with the aim of examining a depressive phenotype) have shown that chronic administration of corticosterone or laboratory stress are associated with reduced eating behavior (Kvarta et al., 2015) or a diminished ability to establish preference for sweets (Mateus-Pinheiro et al., 2014).

A disease in humans characterized by excessive cortisol production is Cushing's syndrome (CS), which provides a unique model of chronic GC exposure (i.e., that is independent of precipitating chronic life stressors that could potentially have additional downstream functional consequences to cloud interpretations). CS is a rare endocrine disorder (1.2–2.4/million/year) characterized by chronic excess endogenous GCs due to an ACTH pituitary adenoma or a cortisol-producing adrenal adenoma (Lacroix et al., 2015); left untreated, CS results in increased mortality and multiple morbidities including obesity, diabetes, hypertension, cardiovascular disease, and overall lower health-related quality of life (Webb et al., 2008; Feelders et al., 2012; Carluccio et al., 2015). Individuals with CS also exhibit persistent neurocognitive impairments, including in memory and learning (Whelan et al., 1980; Martignoni et al., 1992; Mauri et al., 1993; Forget et al., 2000; Starkman et al., 2001, 2003; Leon-Carrion et al., 2009; Michaud et al., 2009; Pereira et al., 2010; Ragnarsson et al., 2012; Resmini et al., 2012); such deficits are quite anticipated in light of the wide distribution of GC receptors in brain areas (e.g., hippocampus, amygdala, and PFC) that are important for these respective executive functions (McEwen et al., 2016). Such deficits (e.g., in verbal memory) and associated improvement with treatment have been correlated with salivary or urinary cortisol concentrations (Grillon et al., 2004; Hook et al., 2007), supporting the idea that GC abnormalities underlie these deficits. More recently, and of greater importance for the current study, CS patients have also exhibited differences from controls in task-related decision-making (Crespo et al., 2014) and dispositional novelty-seeking (Dimopoulou et al., 2013). Such findings are consistent with a more general role of GCs in healthy individuals in potentially modulating risk-taking behavior during conditions of uncertainty (Coates and Herbert, 2008; Putman et al., 2010).

In the present study, we examined the effects of chronic GC exposure resulting from CS on laboratory food-choice tasks in which participants chose to view high-caloric food images vs. viewing standardized pleasant (e.g., smiling babies), unpleasant (e.g., disfigurement), or neutral (e.g., household objects) images; members of our team originally developed this task for use in cocaine addiction (i.e., evaluating choice for cocaine images) (Moeller et al., 2009). Given data suggesting that, on balance, chronic GC exposure is associated with blunted reward processing, we hypothesized that CS patients would choose to view fewer food-related and/or fewer pleasant-related images than a sample of healthy controls with similar body mass index (BMI). Moreover, given data suggesting that chronic GC exposure drives a metabolic memory that results in long-term mortality risk, cognitive and emotional impairments, and reduced quality of life even after normalization of cortisol levels (Tiemensma et al., 2010a,b; Geer et al., 2012; Pereira et al., 2012; Lambert et al., 2013; Andela et al., 2015; Pivonello et al., 2015), we further hypothesized that such blunted choice, although accentuated in CS participants with active disease, would also be present in CS in remission, who would show improved but not normalized reward processing.

## Methods

### Participants

Participants included 23 patients with CS and 15 overweight healthy controls (OHC) with similar BMI (independent *t*-test: *p* = 0.12), such that results could be more plausibly attributed to differences in GCs between the groups, rather than to more general metabolic disturbances and food-seeking behaviors that often characterize obesity. Of these patients with CS, 13 were pre-surgery with active disease, and 10 were post-surgery with disease in remission; these groups were considered separately in all analyses reported below. All participants provided written informed consent, and the Mount Sinai Institutional Review Board approved the study procedures. Participants were required to be age 17 or older. Enrolled CS participants were those who were eligible for surgery within 3 months of test administration, or who had achieved endocrine remission from previous surgical treatment (range of time since endocrine remission was achieved in the treated cohort was 6–60 months). Exclusion criteria for both groups were pregnancy; untreated hypothyroidism; use of exogenous GCs, in the 1-week prior to testing (for the active CS and OHC cohorts); and being unable or unwilling to comply with the study procedures or give informed consent. Exclusion criteria for the OHC were underlying endocrine or metabolic disorders, history of alcohol abuse, or unstable weight.

Active CS participants had elevated cortisol levels, as measured by 24 h urine free cortisol (UFC) (see also Table 1), and normal or elevated plasma ACTH concentrations (if the participant had Cushing's disease: CD; *N* = 22), or low plasma ACTH (if the participant had adrenal CS; *N* = 1), as previously defined (Nieman et al., 2008). An MRI scan or inferior petrosal sinus sampling, which was indicated for 10 patients, confirmed pituitary source of CD. For CS participants studied after surgical treatment, endocrine remission was confirmed by resolution of CS features, 1–2 day post-operative hypocortisolemia (serum cortisol < 5 mcg/dL) and/or normal 24-h UFC after physiologic oral GC replacement had been discontinued, as previously defined (Nieman et al., 2015). Among all CS patients, two active patients were taking diabetes medications, five active and three remitted patients were taking psychiatric (including anti-anxiety, antidepressant, mood stabilizing, or sedating) medications, and one active patient was taking analgesic medications (for OHC, two participants were taking diabetes medications, and one was taking oral stimulant medication). Four remitted CS patients were taking physiologic oral hydrocortisone replacement (hydrocortisone 15–20 mg daily): two of these patients had required bilateral adrenalectomy in order to achieve endocrine remission after unsuccessful transsphenoidal surgery, and the remaining two were still transiently hypocortisolemic due to successful surgical treatment within the past 6 months. Two remitted patients, who underwent two transsphenoidal surgeries, developed hypopituitarism and were taking pituitary endocrine replacement, including physiologic levothyroxine in both and testosterone in one. All were ambulatory with normal renal function and no liver disease.

**Table 1:** **Demographics, self-report measures, and clinical characteristics by study group**.

	**Statistical test**	**Active Cushing's (*N* = 13)**	**Cushing's in remission (*N* = 10)**	**Overweight controls (*N* = 15)**
Gender (women/men)	$\chi_{\left(2,N\;=\;38\right)}^{2}=$ 3.86	10/3	4/6	7/8
Medication (any) (yes/no)	$\chi_{\left(2,N\;=\;38\right)}^{2}=$ 13.07^*^	12/1^c^	7/3^c^	4/11^a^^,^ ^b^
Age (years)	*F*_(2, 35)_ = 1.09	42.1 ± 12.9	38.4 ± 16.5	34.4 ± 12.4
Race (White/non-White)	$\chi_{\left(2,N\;=\;38\right)}^{2}=7.90$	12/1	10/0	9/6
Ethnicity (non-Hispanic, Hispanic)	χ^2^(2, *N* = 38) = 1.23	11/2	9/1	11/4
Body mass index (kg/m^2^)	$F_{\left(2,\;35\right)}=4.85^{*}$	33.1 ± 4.6^b^^,^ ^c^	28.7 ± 2.9^a^	28.9 ± 4.2^a^
Barrett impulsiveness scale	*F*_(2, 35)_ = 0.28	58.9 ± 8.4	62.0 ± 10.7	59.7 ± 10.9
Childhood trauma questionnaire	*F*_(2, 35)_ = 0.53	40.3 ± 9.3	39.3 ± 15.5	45.5 ± 21.7
Perceived stress scale	$F_{\left(2,\;35\right)}=6.68^{*}$	47.0 ± 8.5^b^^,^ ^c^	36.5 ± 8.4^a^	36.4 ± 8.4^a^
Beck depression inventory	$F_{\left(2,34\right)}=8.78^{*}$	23.7 ± 11.6^b^^,^ ^c^	12.2 ± 12.3^a^	6.7 ± 8.3^a^
State-trait anxiety inventory: state	$F_{\left(2,\;34\right)}=6.65^{*}$	30.5 ± 14.8^b^^,^ ^c^	16.0 ± 15.6^a^	12.9 ± 9.8^a^
State-trait anxiety inventory: trait	$F_{\left(2,\;34\right)}=8.50^{*}$	33.5 ± 12.9^b^^,^ ^c^	15.7 ± 14.7^a^	15.1 ± 11.6^a^
State food craving	*F*_(2, 35)_ = 0.14	42.4 ± 14.6	45.1 ± 11.2	43.1 ± 10.9
Trait food craving	$F_{\left(2,\;35\right)}=4.75^{*}$	136.8 ± 32.8^c^	114.9 ± 42.5	101.4 ± 15.4^a^
Urine free cortisol (μg/24 h)^d^	$F_{\left(2,21\right)}=8.55^{*}$	279.0 ± 172.3^b^^,^ ^c^	54.7 ± 45.8^a^	81.0 ± 25.7^a^
Urine cortisone (μg/24 h)^d^	$F_{\left(2,\;21\right)}=6.31^{*}$	380.0 ± 210.5^c^	216.7 ± 146.1	140.7 ± 51.3^a^

*Numbers are mean ± standard deviation*.

* *omnibus p < 0.05, with significant follow-up pairwise comparisons*.

a *Differs significantly from active Cushing's patients*.

b *Differs significantly from Cushing's patients in remission*.

c *Differs from overweight controls*.

d *Sample sizes are 11, 3, and 10 for active Cushing's, Cushing's in remission, and overweight controls, respectively*.

Table 1 provides demographics, self-reports, and clinical characteristics of the study sample, split by disease status (active CS, CS in remission, OHC). The groups did not differ on gender, age, race, childhood trauma, or trait impulsivity (Patton et al., 1995). However, there was a higher proportion medication usage in all CS patients than controls. Other group differences were between active CS patients vs. the other two groups: active CS patients had a higher BMI and more dysphoric symptoms, including higher scores on the Beck Depression Inventory (BDI) (Beck, 1996), the State-Trait Anxiety Inventory (STAI) (Spielberger et al., 1983), and the Perceived Stress Scale (PSS) (Cohen et al., 1983); such dysphoric symptoms are consistent with neuropsychiatric comorbidities commonly reported in this population (Pereira et al., 2010; Pivonello et al., 2015).

### Picture choice tasks

Two picture choice tasks, which have been previously validated in cocaine addiction as extensively described elsewhere (Moeller et al., 2009, 2010, 2012a,b, 2013, 2014), were completed by all study participants at the Mount Sinai Clinical Research Unit in the morning after an overnight fast ≥8 h duration. The tasks—one with explicit contingencies and one with probabilistic contingencies—use standardized pleasant, unpleasant, and neutral images selected from the International Affective Picture System (IAPS) (Lang et al., 2008). For the current study, we incorporated images of food in lieu of cocaine, matched on size and ratio of human to non-human content. These food images depicted palatable “junk” foods (e.g., hamburgers, pizza, or ice cream), and people eating these foods. These two tasks assess complementary notions of choice as described below. Table 2 provides the raw means and standard deviations of both choice tasks by study group.

**Table 2:** **Descriptive statistics of choice behavior by study group**.

	**Active Cushing's (*N* = 13)**	**Cushing's in remission (*N* = 10)**	**Overweight controls (*N* = 15)**
**EXPLICIT TASK (TOTAL BUTTON PRESSES)**			
A. Pleasant pictures	170.9±147.1	243.8±205.8	186.9±135.2
B. Unpleasant pictures	6.9±8.5	4.3±8.9	36.3±61.9
C. Neutral pictures	127.4±129.6	147.9±128.1	140.3±104.2
D. Food pictures	132.3±111.8	195.5±164.2	207.1±157.1
**PROBABILISTIC TASK (TOTAL SELECTIONS)**			
A. Pleasant pictures	20.2±5.8	19.4±5.3	18.5±9.2
B. Unpleasant pictures	11.2±7.6	11.1±7.2	8.3±5.4
C. Neutral pictures	17.5±8.0	16.6±5.7	8.9±6.2
D. Food pictures	18.5±9.4	24.1±5.1	19.5±8.8

*Numbers are mean ± standard deviation, without correction for any covariates*.

#### Explicit choice task (Figure 1A)

Participants chose via continued button pressing between two fully-visible side-by-side images from different picture categories (pleasant, unpleasant, neutral, and food) [we note that a fifth picture category, blank (black) screens containing no image, was also included on this task, but these null images were not analyzed in this study to better equate behavior on the two tasks]. Choice for a desired image enlarged this chosen image to fully cover the screen, which participants could view for the trial duration of 5 s by continued button pressing; 0.5 s of non-response, however, returned the side-by-side image display. After each trial, a new trial with new images ensued. Button pressing (i.e., “working”) for images was an important design feature of this task, meant to model “working” for a desired reward; from a behavioral economic perspective, choice on the explicit task can be thought to index breakpoint (i.e., preference for one category of image over another when there is a cost of effort expenditure) (e.g., Mackillop et al., 2010; Moeller et al., 2013). We summed the number of button presses (across 70 total trials), indexing vigor of responding, per picture category.

**Figure 1:**
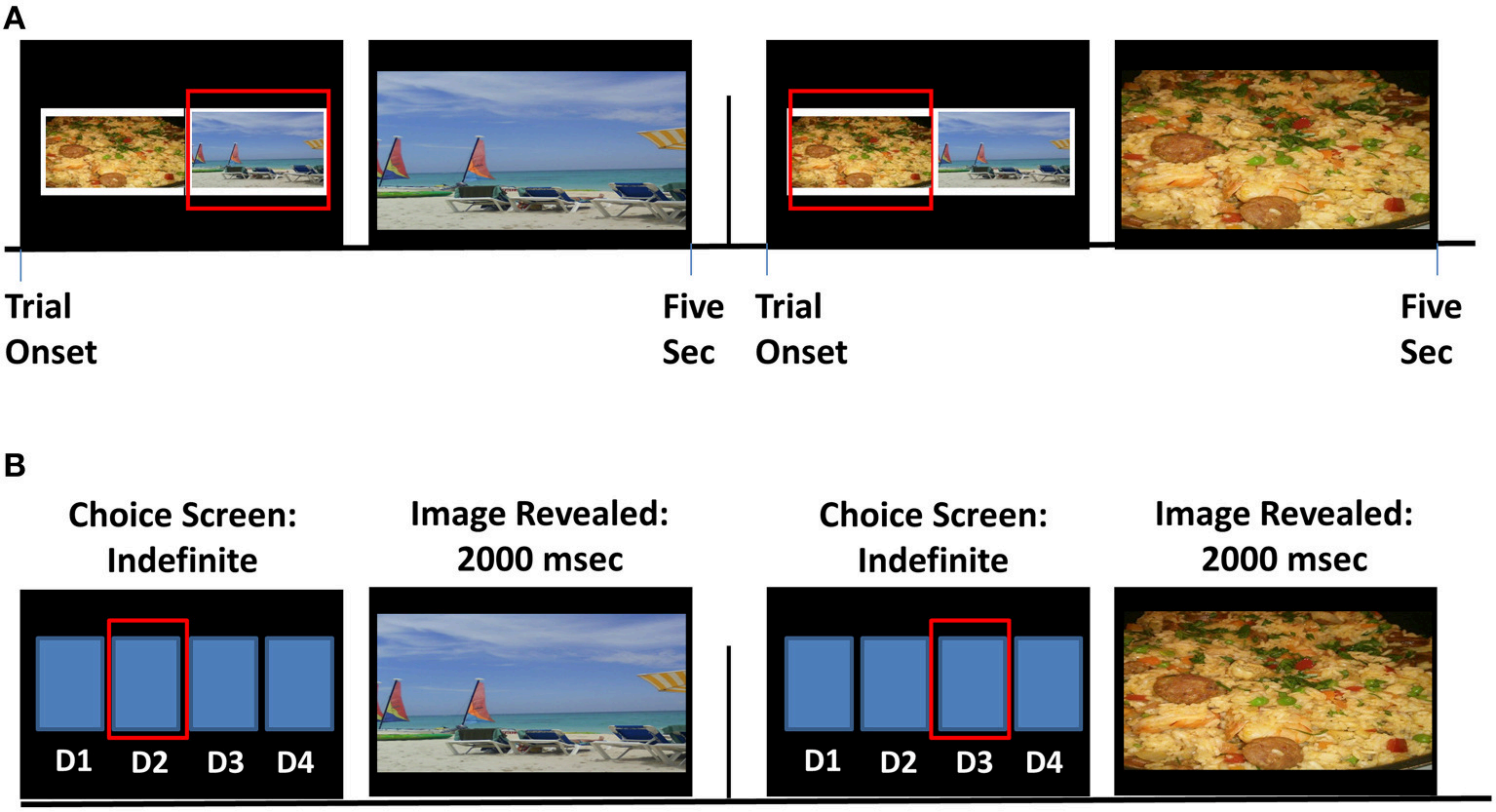
**Task schematics of the explicit and probabilistic food choice tasks. (A)** The explicit task included training and one block, consisting of 70 trials. Two sample trials are displayed. Continuous button pressing for a given image (indicated by the red box) enlarged the corresponding image, for the 5-s trial duration; no response (for 0.5 s) after initial response returned the side-by-side display. **(B)** The probabilistic task included training and four task runs. In each trial within a run, participants pressed one of four buttons corresponding to their chosen deck (D1, D2, D3, or D4; also indicated by the red box).

#### Probabilistic choice task (Figure 1B)

On each trial, participants chose via a single button press to view pictures hidden under flipped-over cards, arranged in four decks. Immediately after choosing from a particular “deck,” an image was revealed that covered the entire screen for 2 s of passive viewing. The images were arranged probabilistically: each deck contained 26 (out of a total of 30) pictures from a particular category (e.g., pleasant), allowing pictures from other categories to be interspersed within each deck (two pictures from a secondary category, e.g., food; and one picture from each of the two remaining categories, e.g., unpleasant and neutral). After participants selected from a particular deck eight total times (corresponding to one task run), deck location of the four picture categories shifted. Thus, this task contained a “seeking” component, rather than a “working” component: throughout, participants needed to seek (and re-seek, once task contingencies changed) their preferred deck(s). In contrast to the explicit task, choice on the probabilistic task was meant to model more standard notions of choice; here, from a behavioral economic perspective, probabilistic choice can be thought to reflect intensity (i.e., preference for one category of image over another when there is no cost associated with the choice) (e.g., Mackillop et al., 2010; Moeller et al., 2013). We summed the total number of cards selected per picture category across four task runs.

### Food craving questionnaires

#### State food craving

Current food craving was measured with the Food Craving Questionnaire-State [FCQ-S (Cepeda-Benito et al., 2000; Nijs et al., 2007)]. The FCQ-S consists of 15 items assessing: desire to eat, anticipation of positive reinforcement from eating, anticipation of negative reinforcement (reduction of negative affect) from eating, lack of control over eating, and (physiological) hunger; higher scores reflect stronger food craving. Participants respond, using a Likert-type scale, how much each item is true of them *right now*. Demonstrating applicability of this questionnaire to the current study, FCQ-S scores were increased after exposure to food images in “high cravers” of chocolate (Meule et al., 2012), and were correlated with increased attention allocation (that interfered with response inhibition) to food vs. neutral pictures (Meule et al., 2014a).

#### Trait food craving

Trait food craving was assessed with the Food Craving Questionnaire-Trait (FCQ-T) (Cepeda-Benito et al., 2000; Nijs et al., 2007). The FCQ-T consists of 39 items assessing: intentions/plans to eat, anticipation of positive reinforcement from eating, anticipation of negative reinforcement from eating, lack of control over eating, preoccupation with food, (physiological) hunger, emotions preceding or following food cravings or eating, environmental cues that may elicit food cravings, negative emotions including guilt experienced as a consequence of food cravings, and/or indulging such cravings; higher scores again indicate higher trait craving. Individuals are asked to respond, using a Likert-type scale, how much each item is true of them *in general*. Unlike the FCQ-S, and appropriately for a trait measure, the FCQ-T was unaffected by food deprivation (Meule et al., 2014b). In the current study, active CS had higher trait (but not state) food craving than OHC (Table 1).

### Urine cortisol and cortisone measures

Twenty-four hour UFC and cortisone were quantified by gas chromatography-mass spectroscopy (GC-MS) as previously described (Yehuda et al., 2009a,b). The limit of sensitivity is 2 ng, the inter-assay coefficients of variations are < 10%, and the normal range for UFC is 10–100 μg/24 h. As to be expected, these measures were highest in active CS (Table 1).

### Statistical analyses

#### Effects of disease status on food picture choice

We analyzed both choice tasks using a 4 (Picture Type: pleasant, unpleasant, neutral, food) × 3 (Disease Status: active CS, CS in remission, OHC) mixed analysis of covariance (ANCOVA), with the total number of presses (explicit task) or selections (probabilistic task) entered as covariates in the appropriate model to control for individual differences in response frequency. Significant interactions were followed by paired (within-group) and independent (between-group) comparisons that similarly controlled for response frequency. For the latter, a core interest was in testing for between-group linear contrasts (i.e., stepwise patterns in the effects as a function of Disease Status). In all ANCOVAs and follow-up comparisons, *p* < 0.05 was considered significant.

#### Correlation analyses

For both tasks, we examined associations between the food choice variables and the food vs. pleasant choice difference scores (food > pleasant) with the variables of interest specified below. The focus on this specific food > pleasant difference score follows from our prior results in individuals with cocaine use disorder, in which cocaine vs. pleasant (cocaine > pleasant) choice was particularly useful in predicting drug-relevant outcomes (Moeller et al., 2012a, 2013). Correlations were conducted across all participants and split by the three study groups. Due to the presence of some outliers across various measures, correlations were conducted using non-parametric (Spearman) analyses. In all correlational analyses, *p* < 0.01 was considered significant to protect against Type I error. After satisfying this initial criterion, however, we retained significant correlations if they achieved a significance level of *p* < 0.05 when accounting for covariates.

##### Between-task reliability and initial validity

Because these tasks were new to a CS population, and because they included a new image category (food), we tested for intercorrelations between the explicit and probabilistic task scores. To provide evidence of construct validity of these tasks in CS, we also examined correlations between the task variables with BMI and self-reported food craving (state and trait).

##### Associations with cortisol levels

To provide further attribution of our effects to GCs, we performed correlations between the two food-choice variables and the two food > pleasant choice scores with urine markers of cortisol inclusive of free cortisol and cortisone in the subsample of participants for whom these data were available.

#### Effects of covariates

Because medication use, BMI, and dysphoric symptoms (i.e., state and trait anxiety, perceived stress, and depression symptoms) differed between the groups (Table 1), these variables were covaried in subsequent ANCOVAs or multiple regressions as appropriate. Note that food craving and urine cortisol/cortisone were considered dependent variables, not covariates.

## Results

### Effects of disease status on food picture choice

**Figure 2:**
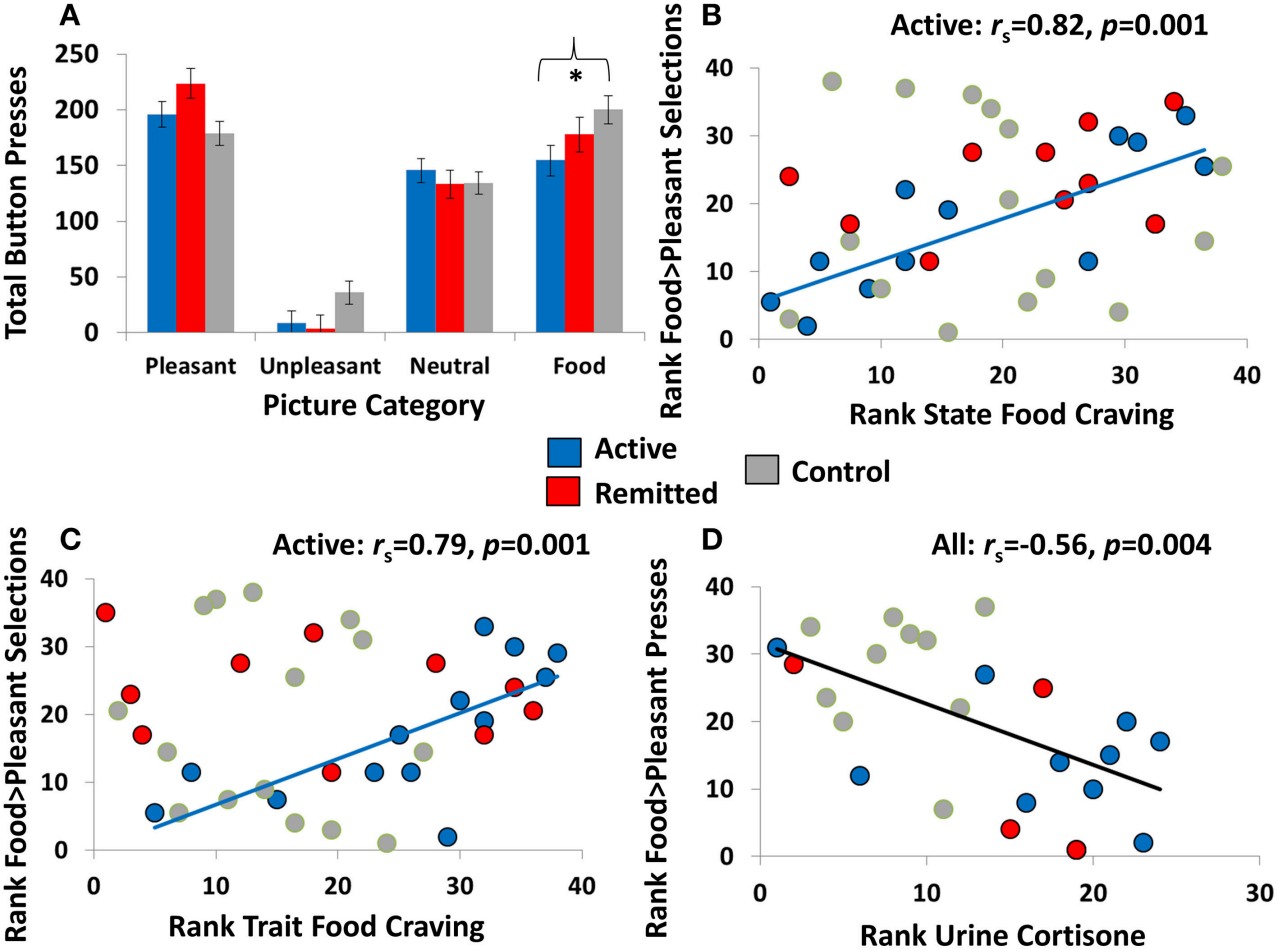
**Relevant choice task results and scatterplots showing associations between food-related choice with state food craving and cortisol. (A)** Results of the explicit task showing total button presses (estimated marginal means) for each of the four picture categories (pleasant, unpleasant, neutral, and food) for individuals with active Cushing's syndrome (*N* = 13), individuals with Cushing's syndrome in remission (*N* = 10), and overweight comparison participants (*N* = 15). The asterisk indicates a significant linear contrast among the groups at *p* < 0.05, and error bars represent standard error of the mean. Note that results of the probabilistic choice are not shown, given the nonsignificant Picture Category × Diagnosis interactions (for descriptive statistics of this task, see Table 2). **(B,C)** In individuals with active Cushing's syndrome but not the other two study groups, higher probabilistic food choice (compared with probabilistic non-food pleasant choice) correlated with higher state and trait food craving. **(D)** Across all participants for whom urine cortisol markers were available (*N* = 24), higher explicit food choice (compared with explicit non-food pleasant choice) correlated with lower urine cortisone.

Results of the mixed 4 (Picture Type) × 3 (Disease Status) ANCOVAs revealed main effects of Picture Type [*F*_(3, 32)_ > 36.50, *p* < 0.001]: on both tasks, unpleasant choice was lowest, followed by neutral choice, and these were both lower than pleasant and food choice (all pairwise comparisons, *p* < 0.01). There were no main effects of Disease Status (both *p* > 0.096). Of greater interest, on the explicit task, the Picture Type × Disease Status interaction reached significance [*F*_(6, 64)_ = 3.12, *p* = 0.010] (Figure 2A). Planned linear contrast analyses showed that active CS participants made fewer food choices than CS participants in remission, who in turn made fewer food choices than OHC (*p* = 0.021); linear contrasts for the other picture categories did not reach significance (all *p* > 0.074), indicating specificity to food choice. Follow-up within-group comparisons showed that both active CS and CS in remission pressed for fewer food pictures than pleasant pictures (both *p* < 0.012) but not neutral pictures (both *p* > 0.063). OHC showed an opposite pattern of results, pressing for food pictures more than neutral pictures (*p* = 0.003) but not pleasant pictures (*p* = 0.099). These collective analyses suggest that CS patients displayed reduced vigor for responding to food reward, particularly in those with active disease. Because the Picture Type × Disease Status interaction did not reach significance on the probabilistic task [*F*_(6, 64)_ = 1.18, *p* = 0.34], no follow-up comparisons were performed.

### Correlation analyses

#### Between-task reliability and initial validity

Correlations did not reach significance at *p* < 0.01 across the whole sample. Nevertheless, after splitting the three groups, a positive correlation emerged between the food > pleasant choice variables on the two tasks in OHC (*r*_s_ = 0.74, *p* = 0.001) but not in either of the CS groups (*r*_s_ < 0.39, *p* > 0.19). In addition, higher food > pleasant choice, this time on the probabilistic task, positively correlated with higher state (*r*_s_ = 0.82, *p* = 0.001) and trait (*r*_s_ = 0.79, *p* = 0.001) food craving in active CS but not in CS in remission or OHC (*r*_s_ < |0.38|, *p* > 0.28) (Figures 2B,C). This correlation suggests that food-related choice on this task is a valid metric of the desire to eat, both currently and in general, in a CS patient population with active disease.

#### Associations with cortisol levels

Across all participants, food > pleasant choice on the explicit task negatively correlated with urine free cortisone (the higher the cortisone, the lower the food-related choice) (*r*_*s*_ = −0.56, *p* = 0.004) (Figure 2D). A similar trend was observed for UFC (*r*_*s*_ = −0.42, *p* = 0.041), which, although not reaching nominal significance, increases confidence in the cortisone effect.

### Effects of covariates

The Picture Type × Disease Status interaction remained significant after controlling for medication use (*p* = 0.048), BMI (*p* = 0.018), and dysphoric symptoms (state and trait anxiety, perceived stress, and depression symptoms: *p* < 0.022). In addition, results of multiple regression analyses controlling for these same covariates showed that correlations were still detected between the food > pleasant scores on both tasks in OHC (*p* < 0.004), between food > pleasant probabilistic choice with state (*p* < 0.007) and trait (*p* < 0.032) food craving in active CS, and between food > pleasant explicit choice and urine cortisone in all participants (*p* < 0.025). Thus, it is unlikely that these potential confounds drove the current results.

## Discussion

In this study, CS with active disease, CS with disease in remission, and OHC participants completed two behavioral tasks that tested the choice to view food pictures vs. non-food-related pleasant, unpleasant, and neutral pictures. We hypothesized that, as a model of chronic exposure to excessive GCs, individuals with CS (and especially those individuals with active disease) would make fewer food-related choices (and fewer pleasant-related choices) than OHC. Results supported the hypothesis that individuals with higher endogenous GC levels, independent of BMI, exhibited decreased food (but not pleasant) choice on the explicit (but not probabilistic) task.

The primary result of this study was that, when task contingencies were explicit (i.e., fully certain) and choice required working (continuously pressing buttons) to view the respective stimuli, individuals with active CS chose to view fewer food images than individuals with CS in remission, who in turn chose to view fewer food images than OHC; thus, CS patients, especially those with active disease, were less apt to *exert effort* to view food cues. Although at first blush this decreased food choice may be unexpected given that individuals exposed to chronic stress or GCs often consume more calories and/or eat less healthfully (Tomiyama et al., 2011; Groesz et al., 2012; Hoffman et al., 2012; Talbot et al., 2013; Aschbacher et al., 2014), it is critical to note that the “control” group was also overweight (i.e., not differing from the overall CS group on BMI, which was a goal in our recruitment; see Methods). Moreover, our results are consistent with prior work showing that chronic excessive exposure to GCs, here attributable to active CS but potentially also generalizable to chronic stress or chronic GC administration, is associated with blunted reward sensitivity in human- (Dillon et al., 2009; Goff et al., 2013) and animal models (Mateus-Pinheiro et al., 2014; Kvarta et al., 2015). Because in the current study results were specific to the food-choice task that required “working,” it is possible that active CS patients have a higher threshold for perceiving food as reinforcing, potentially needing to consume more to achieve the same hedonic effect. An important parallel can be made to addiction literature, whereby reduced drug sensitivity is associated with increased or uncontrolled drug use [e.g., whether because of putatively drug-mediated adaptations to dopamine neurotransmission in drug addiction (Volkow et al., 1997, 2010; Martinez et al., 2009; Peechatka et al., 2015) or because of genetics that elevate susceptibility to developing addiction later in life (Edenberg, 2007; de Wit and Phillips, 2012; Gubner et al., 2013)]. More broadly, our results support and extend prior research in CS on deficits in memory, learning, and decision-making (Whelan et al., 1980; Martignoni et al., 1992; Mauri et al., 1993; Forget et al., 2000; Starkman et al., 2001, 2003; Leon-Carrion et al., 2009; Michaud et al., 2009; Pereira et al., 2010; Ragnarsson et al., 2012; Resmini et al., 2012; Crespo et al., 2014); and that such neurocognitive deficits may be ameliorated, but not fully normalized, even after surgical remission (Tiemensma et al., 2010a,b; Geer et al., 2012; Pereira et al., 2012; Lambert et al., 2013; Andela et al., 2015; Pivonello et al., 2015).

In further analyses, we correlated our behavioral choice results with self-reported food craving and *in vivo* GC levels; these analyses, respectively, were meant to validate these food-choice tasks as a model of food reward in a CS population and to provide a putative neurobiological correlate of the food-choice results. For the latter (neurobiological correlate), and corroborating our main between-group analyses above, correlations emerged between food-related choice on the explicit task and urine GCs: across all participants, 24-h urine cortisone correlated with explicit food-related choice, with similar albeit nonsignificant effects emerging for UFC. The underlying mechanism of these effects, which requires testing in further studies, could involve GC modulation of the dopamine system that alters the incentive salience of food cues (Borges et al., 2013; Yau and Potenza, 2013; Soares-Cunha et al., 2014). For the former (task validity), probabilistic (though not explicit) food-related choice positively correlated with state and trait food craving within active CS. Thus, although between-group effects in the current study were not observed on this probabilistic task, correlations with food craving help to validate these food-choice tasks as a model of food reward in active CS.

Limitations of this study include the following. First, the current study did not include a sample of normal-weight controls, and without this reference group we cannot definitively conclude whether results are primarily driven by decreased food-choice in CS, increased food-choice in OHC, or both. Future studies will need to include a normal-weight control group. Nevertheless, our paramount concern in this initial study was to equate the groups on BMI, which indeed was achieved for the full CS sample. Although a BMI group difference emerged when splitting the groups by disease status (Table 1), it is important to note that our results were robust to statistical correction for BMI, indicating that this variable did not drive our results. Second, urine cortisol markers were unavailable in half the study sample. However, effects with cortisone were indeed observed using a relatively strict statistical threshold, reducing the potential for spurious effects; and, more importantly, in our main analyses we found stepwise effects as a function of remission status on behavioral choice in the entire sample. Third, we did not obtain self-report ratings of preference for the food stimuli prior to their use in the current tasks. However, main effects of Picture Type indicated that these food images were indeed palatable, such that all participants chose them for viewing more often than neutral images or unpleasant images. Fourth, it is possible that demand characteristics or participants' task-related motivation could have contributed to the lower food-related choice in CS participants. However, if results were driven by demand characteristics associated with the desire to reduce food intake and lose weight, we strongly suspect that OHC participants would have been similarly motivated to avoid choosing food images. If results were driven by reduced task-related motivation in CS (e.g., due to anhedonia), one could anticipate that choice for non-food-related rewarding stimuli (here, pleasant images) would be reduced as well (Pizzagalli et al., 2009; Domschke et al., 2015; Fletcher et al., 2015), but this pattern of effects did not occur; moreover, dysphoric symptoms did not explain our findings.

In conclusion, chronic GC exposure from active CS was associated with reduced simulated food choice when compared with such choice in overweight individuals with intact GC functioning; CS patients in remission with remediated GC functioning showed an intermediate pattern of food choice, suggesting that that prior GC exposure may exert lasting effects on brain reward systems. Future studies can use neuroimaging and/or neurochemical approaches in CS, OHC, and (importantly) normal-weight controls to test for commonalities and/or differences in food choice and its neurobiological underpinnings among these study groups. Longitudinal designs can also test whether GC-mediated food-choices have downstream consequences, such as increasing risk for weight gain and/or cardiovascular events. More broadly, beyond illuminating neurocognitive sequelae of CS (and possibly chronic stress), our findings can also inform the neurobiology of elevated and/or dysregulated cortisol as seen in aging, major depressive disorder, and/or Alzheimer's disease (Pereira et al., 2012; Notarianni, 2013; Gupta and Morley, 2014; Du and Pang, 2015; Furtado and Katzman, 2015).

## Author contributions

SM, RG, and EG designed research. LC, VC, and YL performed research. SM, IM, NW, RY, and EG analyzed data. SM and EG wrote the paper. All authors provided critical revisions of the paper.

### Conflict of interest statement

The authors declare that the research was conducted in the absence of any commercial or financial relationships that could be construed as a potential conflict of interest.
